# Association between levels of thiamine intake, diabetes, cardiovascular diseases and depression in Korea: a national cross-sectional study

**DOI:** 10.1017/jns.2021.23

**Published:** 2021-04-27

**Authors:** Hai Nguyen Duc, Hojin Oh, In Mo Yoon, Min-Sun Kim

**Affiliations:** 1 Department of Pharmacy, College of Pharmacy and Research Institute of Life and Pharmaceutical Sciences, Sunchon National University, Sunchon, Jeonnam 57922, Republic of Korea; 2 Unimedi Plastic Surgery Clinic, Suite 302, 3rd floor, Nonhyeon-ro 833, Sinsa-dong, Gangnam-gu, Seoul 06032, Republic of Korea

**Keywords:** KNHANES, Mental health, Non-communicable diseases, Thiamine intake

## Abstract

The present study aimed to determine thiamine intake levels and the association between thiamine intake, diabetes, cardiovascular diseases and mental health. Participants were interviewed to obtain data on socio-demographic characteristics, lifestyle, current medications, medical and family history. The daily intake of thiamine was assessed by a 24-h recall. The mean age of the 34 700 study subjects was 42⋅9 years (sd 22⋅8, min–max: 1–80) and 19 342 (55⋅7 %) were women. The levels of thiamine intake were 1⋅126 mg (2016), 1⋅115 mg (2017) and 1⋅087 mg (2018) for women, which were equal to or only slightly above the recommended intake of 1⋅10 mg/d for women. The levels of thiamine intake from 2014–15 and 2016–18 significantly decreased. The estimated percentage of insufficient thiamine intake was 37⋅8 % (95 % CI 37⋅3, 38⋅4). Multivariable regression analysis adjusted for potential confounders showed that thiamine intake was critically associated with lower risks of hypertension, MI or angina, type 2 diabetes, depression and dyslipidemia. The daily thiamine intake from food can reversal the risks of hypertension (OR 0⋅95; 95 % CI 0⋅90, 0⋅99), MI or angina (OR 0⋅84; 95 % CI 0⋅74, 0⋅95), type 2 diabetes (OR 0⋅86; 95 % CI 0⋅81, 0⋅93), depression (OR 0⋅90; 95 % CI 0⋅83, 0⋅97) and dyslipidemia (OR 0⋅90; 95 % CI 0⋅86, 0⋅95), respectively. Further works are needed to identify the effects of thiamine and non-communicable diseases (NCDs) and mental health. A preventive thiamine supplementation strategy should be adopted to target NCDs and mental health and risk factors associated with thiamine deficiency. The optimisation of NCD control and mental health protection is also a vital integral part of Korea's public health system.

## Introduction

Over the last decade, rapid changes in diets and lifestyles that have taken place due to urbanisation, industrialisation, economic development and globalisation, and these have changed dietary patterns towards high-fat, high-saturated and low-energy-dense diet^(1,2)^. However, inappropriate dietary patterns, reduced physical activities, increased tobacco use and the resulting increase of diet-related chronic diseases have increased in concert with living standards and food availability. Furthermore, non-communicable diseases (NCDs) such as obesity, hypertension, diabetes mellitus, cardiovascular diseases (CVDs), stroke and cancers are increasingly becoming causes of disability and premature death, which has placed extra pressure on the national health budget. Of note, in developing and developed countries, CVDs are the main cause of morbidity and mortality and are directly associated with an imbalanced diet^(3)^.

Recent studies have expanded to understanding of the effects of diet on morbidity and premature deaths due to NCDs^(4)^. Thiamine, also referred to as vitamin B1, is a water-soluble vitamin and a coenzyme in the metabolism of carbohydrates and branched-chain amino acids^(5)^. Thiamine is a crucial micronutrient and can be obtained from different food sources, but its serum levels can be affected by many factors, such as high temperatures and pH, diuretics, high-calorie diet containing simple carbohydrates, chronic alcoholism, fever, excessive exercise, pregnancy and lactation, stress and trauma^(6–9)^. Furthermore, the half-life of thiamine in the body ranges only 1–3 weeks^(8)^. These factors do much to explain why thiamine inadequate diet commonly results in thiamine deficiency in developed countries. Increasing evidence shows that thiamine deficiency is directly or indirectly associated with many CVDs and diabetes, obesity, dyslipidemia, angina, myocardial infarction (MI) and mental health (e.g. depression)^(10)^. However, the relationships between thiamine intake and CVDs, diabetes, dyslipidemia and mental health are still unclear. In the present study, we used data collected during national population-based surveys to determine thiamine intake levels and to examine the associations of thiamine levels and cardiovascular disease, diabetes, dyslipidemia and mental health.

## Methods

### Study population

We used a multi-stage, stratified, cluster-sampling procedure that regarded the geographic area, the level of urbanisation, economic growth status, and gender and age distribution, derived from the Korean National Health and Nutrition Examination Survey (KNHANES) conducted by the Korean Ministry of Health and Welfare, specifically the KNHANES VI (2014–15) and the KNHANES VII (2016–18)^(11)^. Each survey includes a new sample of about 8000 individuals from non-institutionalised Korean citizens who are representative of the population. The individuals surveyed were randomly selected from 7550 households (2014), 7380 (2015), 8150 (2016), 8127 (2017) and 7992 (2018). In the present study, participants who (1) have fully take part in three parts including a health interview survey, a health examination survey and a nutrition survey, (2) provided information on nutrients that contribute to thiamine intake. Of the 39 199 participants who participated in KNHANES surveys from 2014 to 2018, we excluded 4499 records missing thiamine intakes. Thus, the data of 34 700 participants were subjected to analysis. Written informed consent was required for both patients and family members; parental informed consent was obtained on behalf of all minors before examinations, which were performed by the Health and Nutrition Examination Department of the Korea Centers for Disease Control and Prevention. The present study was conducted according to the guidelines laid down in the Declaration of Helsinki and all procedures involving human subjects/patients were approved by the KNHANES inquiry commission and the Institutional Review Board of Sunchon National University. All methods were performed following a standardised protocol. Detailed information on the plan, standardised protocol and licence of these surveys was available on the KNHANES Web site (http://knhanes.cdc.go.kr/).

### Laboratory measurements

Blood samples were collected after ≥8-h fasting and were analysed at Neodin Medical Institute in Korea. The levels of total cholesterol, high-density lipoprotein cholesterol (HDL-C), triacylglycerols, low-density lipoprotein cholesterol (LDL-C) and fasting glucose were then measured by an enzymatic assay using a Hitachi automatic analyzer 7600 (Hitachi, Tokyo, Japan). Serum low-density lipoprotein cholesterol (LDL-C) is calculated using the Friedewald equation: serum LDL-C ¼ serum total cholesterol − serum HDL-C − serum triacylglycerol/5.

### Parameters

We used the database of the KNHANES health interview survey to classify the demographic status and healthy lifestyles. During medical checkups, the height, weight, waist circumstance and blood pressure were measured using the standard procedure. Body mass index (BMI) (kg/m^2^) is calculated using the formula: BMI = weight (kg)/height^2^ (m^2^). Waist circumstance (cm) was measured at the midpoint between the bottom of the rib cage and the iliac crest of the mid-axillary line when exhaling. Blood pressure was measured three times with intervals of 5 min using a mercury sphygmomanometer with a subject seated after a 5-min stabilisation period. Final blood pressure was the average of the second and third measurements. Education level was classified as below middle school, high school and college or higher. Residence areas were classified into urban and rural. Occupations were classified as follows: (1) managers and professional, (2) office and clerical workers, (3) service and sales workers, (4) agriculture, forestry and fishing workers, (5) craft, plant and machine operators and assemblers, (6) elementary occupations and (7) unemployed. Monthly house incomes were classified as <2000, 2000–4000, 4000–6000 and ≥6000 thousand won. Alcohol intakes were classified as low and high (high-risk drinking was defined as >5 drinks per day and ≥1 month). Subjects with a lifetime history of smoking of >100 cigarettes in their lifetime and still smoked daily or occasionally were classified as current smokers; others were classified as ex/non-smokers. Physical activity was dichotomised as regular or irregular. Regular physical activity was defined as follows: (1) vigorous physical activity (running, climbing, fast cycling, fast swimming, football, basketball, squash, singles tennis, rope jumping or occupational or recreational activity involving the carrying of heavy objects), ≥20 min per session ≥3 d/week; (2) moderate physical activity (slow swimming, doubles tennis, volleyball, or occupational or recreational activity involving the carrying of light objects); ≥30 min per session ≥5 d/week and (3) walking; ≥30 min per session ≥5 d/week. A family history of cardiovascular disease was defined as having at least one parent or sibling with a diagnosis of hypertension, ischemic heart disease or stroke. A family history of type 2 diabetes and hyperlipidemia was defined as having at least one parent or sibling with a diagnosis of type 2 diabetes and hyperlipidemia.

### Diabetes, cardiovascular diseases and mental health

Dyslipidemia was defined as one or more of the following: LDL-C ≥160 mg/dl, triacylglycerol ≥200 mg/dl and HDL-C <40 mg/dl. Hypertension was defined as having either systolic blood pressure (SBP) ≥ 140 mmHg or diastolic blood pressure ≥ 90 mmHg or on anti-hypertensive medication. Type 2 diabetes mellitus was defined as having a fasting plasma glucose ≥126 mg/dl or on anti-diabetic medication, or HbA1c ≥ 6⋅5 %. Stroke, angina, MI, MI or angina were defined as physician diagnosis, the current presence or treatment for stroke, angina, MI, MI or angina. Variables that were taken into account were the self-perception of stress and depression. Participants were asked how much stress they encountered in their everyday routine for self-perception of stress. They were asked to identify stress levels, including very often, sometimes, rarely and a few times or never. Stress was defined if participants answer very often or sometimes. Depression was defined as physician diagnosis, the current presence or treatment for depression.

### Thiamine intake

Daily food intake was determined using the 24-h recall method. Before assessing the food intake, all participants were told to report their normal dietary habits. A semi-quantitative questionnaire on food frequency, which addressed the intakes of sixty-three food products, was completed for each participant. The frequency of food intake was measured using nine classifications: ‘never or rarely’, ‘once a month’, ‘two to three times a month’, ‘one to two times a week’, ‘three to four times a week’, ‘five to six times a week’, ‘once a day’, ‘twice a day’ and ‘three or more times every day’. The daily total energy intakes were measured using Estimated Energy Requirement (EER) in Korea^(12)^. Nutrient intake was calculated using the Can-Pro Ver 3.0 nutrient intake assessment software developed by the Korean Nutrition Society. In the present study, sufficient daily thiamine intakes were defined as ≥1⋅22 mg/d for men and ≥1⋅03 mg/d for women^(13)^. Furthermore, we weighted calorie-adjusted thiamine with a minimum of 0⋅33 mg of thiamine for every 1000 kilocalories (kcal)^(14)^.

### Statistical analysis

All statistical analyses were undertaken using STATA software (version 16.0; StataCorp, Texas, USA). The baseline characteristics of participants were summarised using frequency and proportion for categorical variables; mean and standard deviation or median and interquartile range for continuous variables. Therefore, Student's *t* test for continuous variables and *χ*² test for categorical variables.

The association between thiamine levels and cardiovascular disease, diabetes, dyslipidemia and mental health was examined by logistic regression in adults ≥18 years. Potential covariates were first recognised in the existing literature, or subjective prior knowledge plus those variables with *P*-values of ≤0⋅25 in univariate analysis and were entered in the full model including thiamine (mg), energy (kcal), calorie-adjusted thiamine (mg), age group (<29, 30–39, 40–49, 50–59, 60–69, 70–79 and >80), sex, residential area (rural *v.* urban), marital status (married, living alone), the education level (≤middle school, high school and ≥college), monthly household income (<2000, ≥2000 and <4000, ≥4000 and <6000, ≥6000), smoking status (current smoker, non/ex-smoker), high-risk drinking (yes, no), physical activity (not regular, regular), BMI groups (<18⋅5, ≥18⋅5 and <25, ≥25 and <30, ≥30), and the presence of hypertension, hyperlipidemia and type 2 diabetes^(15)^. In multivariable analysis, backward elimination was used. Any variable which had a *P*-value > 0⋅05 was removed from the model. A log-likelihood ratio test was carried out to compare the ‘bigger’ and ‘reduced’ models. If the log-likelihood ratio test provided a *P*-value of ≤0⋅05, the corresponding variable was retained in the model. The process was repeated till no other variables in the model yielded *P*-values of >0⋅05 (shown in the Supplementary material). To visualise the moderating effect of thiamine, marginal effect analysis was carried out using the results of Poisson regression analysis. Statistical tests were two-sided, *P*-value < 0⋅05 was considered statistically significant.

## Results

In total, 34 700 individuals who participated in the KNHANES 2014–18 survey were included in the present study. The mean age of participants was 42⋅9 years (sd 22⋅8, min–max: 1–80), 19 342 (55⋅7 %) were women. The insufficient and sufficient thiamine intake groups were significantly different in terms of sex, age, married status, occupation, education level, monthly household income, BMI level, waist circumference, elevated cholesterol, reduced HDL-C, elevated HbA1c, elevated glucose, energy intake, haemoglobin, smoking status, drinking status, physical activity, SBP and diastolic blood pressure. Total cholesterol level, HbA1c and fasting glucose were lower in the sufficient thiamine intake group than in the insufficient thiamine intake group. However, dietary energy intake was higher in the sufficient intake group (1369⋅4 (567⋅3) *v.* 2253⋅6 (904⋅6) kcal, *P* < 0⋅001; Table 1).

**Table 1.** Demographic distribution of participants in Korea from 2014 to 2018

Variables	No. of subjects	Thiamine intake		*P*-value
		Insufficiency	Sufficiency	
Sex (%)	34 700			<0⋅001
Male	15 358	5210 (39⋅7)	10 148 (47⋅1)	
Female	19 342	7925 (60⋅3)	11 417 (52⋅9)	
Age (year) (mean, sd)^a^	34 700	41⋅8 (25⋅8)	43⋅6 (21⋅3)	<0⋅001
Marital status (%)	34 699			<0⋅001
Married	23 157	8161 (62⋅1)	14 996 (69⋅5)	
Living alone	11 542	4973 (37⋅9)	6569 (30⋅5)	
Residential areas (%)	34 700			0⋅065
Urban	28 209	10 613 (80⋅8)	17 596 (81⋅6)	
Rural	6491	2522 (19⋅2)	3969 (18⋅4)	
Occupation (%)	25 445			<0⋅001
Managers, professional	3250	970 (10⋅8)	2280 (13⋅9)	
Office worker, clerical workers	2397	750 (8⋅3)	1647 (10⋅0)	
Service workers, sales workers	3047	1064 (11⋅9)	1983 (12⋅0)	
Agriculture, forestry and fishing workers	1204	379 (4⋅2)	825 (5⋅0)	
Craft, plant and machine operators and assemblers	2279	608 (6⋅8)	1671 (10⋅2)	
Elementary occupations	2193	772 (8⋅6)	1421 (8⋅6)	
Unemployed	11 075	4436 (49⋅4)	6639 (40⋅3)	
Education level (%)	31 035			<0⋅001
≤Middle school	14 674	6524 (55⋅2)	8150 (42⋅4)	
High school	7871	2677 (22⋅7)	5194 (27⋅0)	
≥College	8490	2609 (22⋅1)	5881 (30⋅6)	
Monthly household income (%)	34 576			<0⋅001
<2000	9142	3887 (29⋅7)	5255 (24⋅5)	
≥2000 and <4000	9620	3461 (26⋅5)	6159 (28⋅6)	
≥4000 and <6000	7905	2879 (22⋅0)	5026 (23⋅4)	
≥6000	7909	2853 (21⋅8)	5056 (23⋅5)	
BMI group (%)	32 694			<0⋅001
<18⋅5	5163	2673 (21⋅7)	2490 (12⋅2)	
≥18⋅5 and <25	18 242	6388 (51⋅7)	11 854 (58⋅3)	
≥25 and <30	7887	2775 (22⋅5)	5112 (25⋅1)	
≥30	1402	511 (4⋅1)	891 (4⋅4)	
Waist circumference (cm) (mean, sd)^a^	32 718	75⋅3 (15⋅6)	79⋅0 (12⋅6)	<0⋅001
Elevated waist circumference (%)				0⋅064
Yes	10 572	3925 (31⋅7)	6 647 (32⋅7)	
No	22 146	8457 (68⋅3)	13 689 (67⋅3)	
Total cholesterol (mg/dl) (mean, sd)^a^	27 219	189⋅1 (38⋅1)	187⋅6 (36⋅3)	0⋅001
Elevated total cholesterol (%)				0⋅02
Yes	9690	3491 (36⋅9)	6199 (34⋅9)	
No	17 529	5983 (63⋅1)	11 546 (65⋅1)	
LDL-C (mg/dl) (mean, sd)^a^	8341	114⋅4 (35⋅1)	113⋅1 (32⋅7)	0⋅109
Elevated LDL-C (%)				0⋅033
Yes	2471	641 (31⋅5)	1830 (29⋅0)	
No	5870	1394 (68⋅5)	4476 (71⋅0)	
Triacylglycerol (mg/dl) (mean, sd)	27 219	130⋅1 (104⋅5)	129⋅9 (104⋅1)	0⋅864
Elevated triacylglycerol (%)				0⋅641
Yes	7357	2577 (27⋅2)	4780 (26⋅9)	
No	19 862	6897 (72⋅8)	12 965 (73⋅1)	
HDL-C (mg/dl) (mean, sd)^a^	27 207	51⋅2 (12⋅6)	51⋅1 (12⋅3)	0⋅343
Reduced HDL-C (%)				<0⋅001
Yes	9097	3379 (35⋅7)	5718 (32⋅2)	
No	18 110	6090 (64⋅3)	12 020 (67⋅8)	
HbA1c (%) (mean, sd)^a^	27 117	5⋅72 (0⋅80)	5⋅68 (0⋅77)	<0⋅001
Elevated HbA1c (%)				0⋅001
Yes	5211	2007 (21⋅3)	3204 (18⋅1)	
No	21 906	7434 (78⋅7)	14 472 (81⋅9)	
Fasting glucose (mg/dl) (mean, sd)	27 218	101⋅1 (24⋅4)	99⋅8 (23⋅2)	<0⋅001
Elevated glucose (%)^a^				0⋅005
Yes	18 123	3269 (34⋅5)	5826 (32⋅8)	
No	9095	6204 (65⋅5)	11 919 (67⋅2)	
C-reactive protein (mg/l) (mean, sd)	21 559	1⋅21 (2⋅04)	1⋅16 (2⋅04)	>0⋅05
Energy intake (kcal) (mean, sd)^a^	34 700	1369⋅4 (567⋅3)	2253⋅6 (904⋅6)	<0⋅001
Hb (g/dl) (mean, sd)	27 118	13⋅8 (1⋅6)	14⋅1 (1⋅6)	<0⋅001
Smoking status (%)	25 317			<0⋅001
Current smoker	4227	1375 (15⋅3)	2852 (17⋅4)	
Non/ex-smoker	21 090	7590 (84⋅7)	13 500 (82⋅6)	
Drinking status (%)	27 535			<0⋅001
Often	5358	1717 (17⋅8)	3641 (20⋅3)	
Occasionally	13 031	4410 (45⋅8)	8621 (48⋅2)	
Never or rarely	9146	3502 (36⋅4)	5644 (31⋅5)	
Physical activity (%)	34 700			<0⋅001
Not regular	28 936	11 367 (86⋅5)	17 569 (81⋅5)	
Regular	5764	1768 (13⋅5)	3996 (18⋅5)	
Systolic blood pressure (mmHg)^a^ (mean, sd)	28 926	119⋅0 (17⋅3)	117⋅2 (16⋅3)	<0⋅001
Diastolic blood pressure (mmHg) (mean, sd)	28 926	73⋅80 (10⋅6)	74⋅0 (10⋅4)	0⋅045
Family history of CVDs (%)	26 850			0⋅562
Yes	12 612	4311 (47⋅2)	8301 (46⋅8)	
No	14 238	4819 (52⋅8)	9419 (53⋅2)	
Family history of type 2 diabetes (%)	26 633			0⋅062
Yes	5964	2094 (23⋅1)	3870 (22⋅1)	
No	20 669	6988 (76⋅9)	13 681 (77⋅9)	
Family history of hyperlipidemia (%)	26 153			0⋅068
Yes	1734	624 (7⋅0)	1110 (6⋅4)	
No	24 419	8262 (93⋅0)	16 157 (93⋅6)	

BMI, Body mass index (kg/m^2^); CVDs, Cardiovascular disease; elevated HbA1c (≥6⋅0 %), reduced HDL-C (<50 mg/dl, female; <40 mg/dl male), elevated LDL-C (≥100 mg/dl), elevated glucose (≥100 mg/dl), elevated total cholesterol (≥200 mg/dl), elevated triacylglycerol (≥150 mg/dl), elevated waist circumstances (≥80 cm, female, ≥90 cm, male); HDL, High-density lipoprotein; LDL-C, low-density lipoprotein cholesterol.

a Two sample *t* test with unequal variances.

**Fig. 1.**
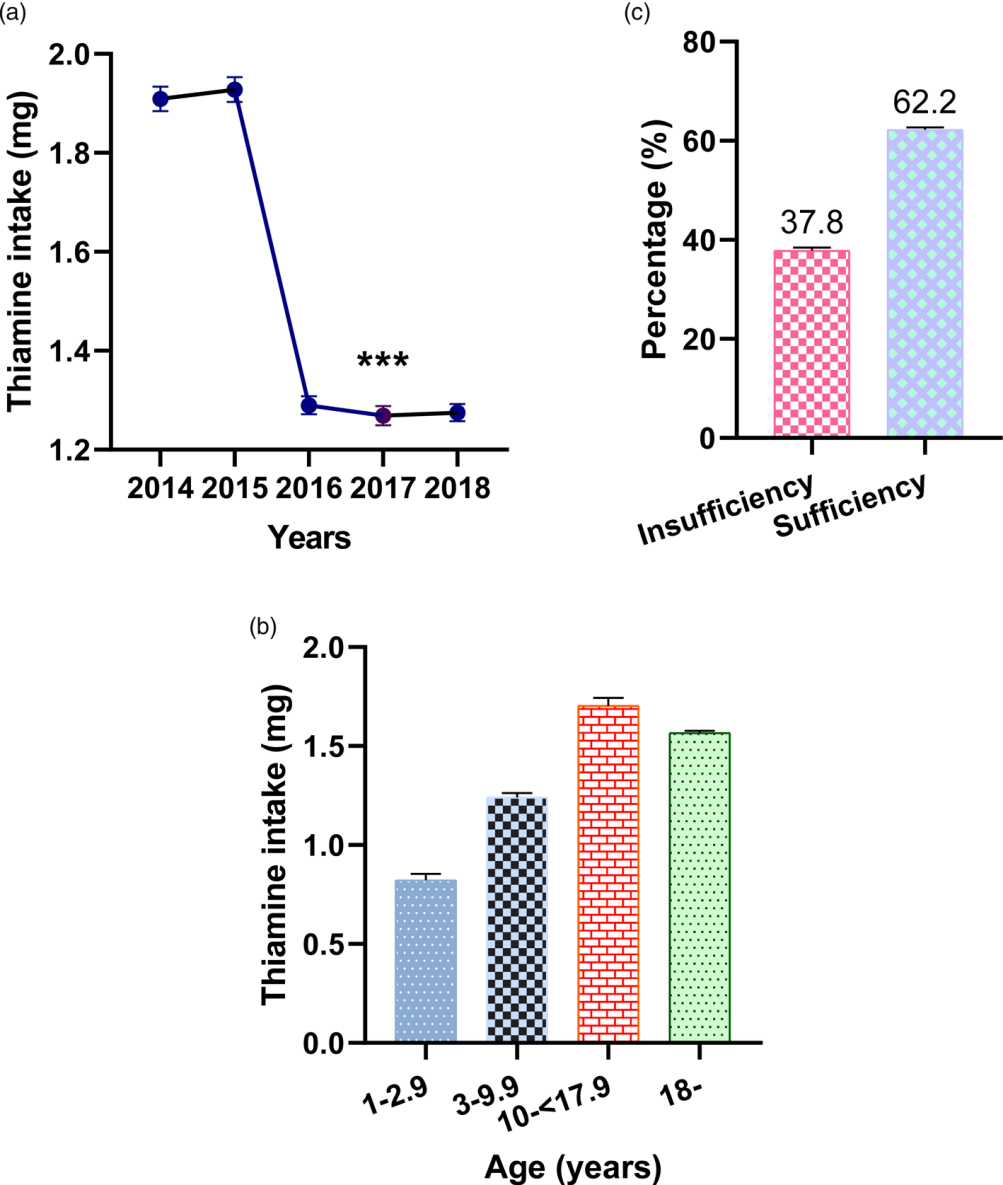
Levels of thiamine intake by year and age group (a and b), and the percentage of insufficient and sufficient thiamine intake among the Korean population (c). ***P < 0⋅001, levels of thiamine intake in each year from 2016 to 2018 compared with 2014 and 2015, one-way ANOVA, Bonferroni. Error bars represent 95 % CIs.

Figure 1 shows thiamine average daily intake levels by year and percentage thiamine intakes among the participants. Average daily thiamine intakes from 2014 to 2018 were 1⋅909 mg (95 % CI 1⋅884, 1⋅934), 1⋅928 mg (95 % CI 1⋅903, 1⋅953), 1⋅290 (95 % CI 1⋅272, 1⋅308), 1⋅269 mg (95 % CI 1⋅250, 1⋅288) and 1⋅275 mg (95 % CI 1⋅258, 1⋅292), respectively. There were significant reductions in daily thiamine intakes between 2014–15 and 2016–18 (*P* < 0⋅001). The estimated percentage of participants with insufficient thiamine intake from 2014 to 2018 was 37⋅8 % (95 % CI 37⋅3, 38⋅4). The average daily intake of thiamine from food was 0⋅82 mg (95 % CI 0⋅79, 0⋅85) for those aged <3 years, 1⋅24 mg (95 % CI 1⋅22, 1⋅26) for those aged 3–10 years, 1⋅70 mg (95 % CI 1⋅66, 1⋅74) for those aged 11–18 years and 1⋅57 mg (95 % CI 1⋅55, 1⋅58) for those aged ≥18.

**Fig. 2.**
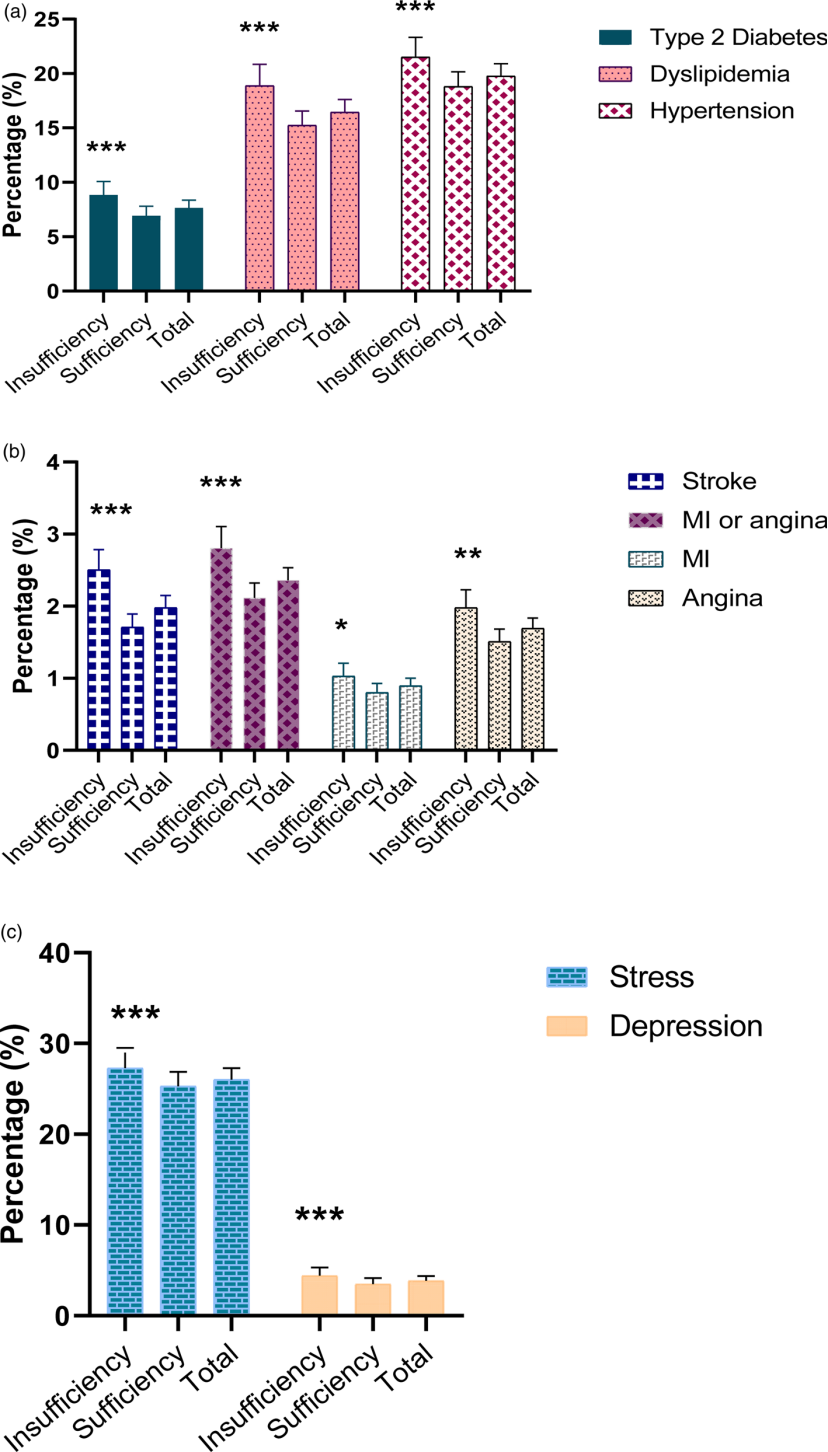
Percentage of type 2 diabetes, dyslipidemia, hypertension (a), and stroke, MI, angina, MI or angina (b) and mental health (c) among the Korean population by thiamine intake level in 2014–18. *P*-value was used by binary logistic regression, ***P < 0⋅001, **P = 0⋅01, *P < 0⋅05 compared with the sufficient thiamine group. Error bars represent 95 % CIs.

Figure 2 shows the percentages of participants with a cardiovascular disease, diabetes or a mental health issue by thiamine intake. The prevalence of hypertension (21⋅5 *v.* 18⋅8 %), stroke (2⋅5 *v*. 1⋅7 %), MI or angina (2⋅8 *v.* 2⋅1 %), MI (1⋅03 *v.* 0⋅8 %), angina (2⋅0 *v.* 1⋅5 %), dyslipidemia (18⋅9 *v.* 15⋅2 %), type 2 diabetes (8⋅8 *v.* 6⋅9 %), depression (4⋅4 *v.* 3⋅5 %) and stress (27⋅3 *v.* 25⋅3 %) was lower in the sufficient than in the insufficient intake group.

Univariable regression analysis showed that the risks of hypertension, type 2 diabetes, dyslipidemia, angina, myocardial infarction, MI or angina, depression and stress were lower in the sufficient group than in the insufficient group (Table S1). Furthermore, thiamine intake is inversely associated with hypertension, MI or angina, type 2 diabetes, depression and dyslipidemia. Multivariable regression analysis adjusted for potential confounders showed that thiamine intake was critically associated with lower risks of hypertension, MI or angina, type 2 diabetes, depression and dyslipidemia. The daily thiamine intake can reversal the risks of hypertension (OR 0⋅95; 95 % CI 0⋅90, 0⋅99), MI or angina (OR 0⋅84; 95 % CI 0⋅74, 0⋅95), type 2 diabetes (OR 0⋅86; 95 %CI 0⋅81, 0⋅93), depression (OR 0⋅90; 95 % CI 0⋅83, 0⋅97) and dyslipidemia (OR 0⋅90; 95 % CI 0⋅86, 0⋅95), respectively (Table 2).

**Table 2.** Odd ratio (95 % confidence interval) for the risk of hypertension, myocardial infarction or angina, dyslipidemia and depression according to the levels of thiamine intake

Variables	Type 2 diabetes (*n* 31 910)	Hypertension (*n* 31 923)	MI or angina (*n* 31 185)	Dyslipidemia (*n* 26 814)	Depression (*n* 31 175)
Unadjusted	0⋅82 (0⋅76–0⋅86)	0⋅86 (0⋅93–0⋅89)	0⋅79 (0⋅72–0⋅87)	0⋅80 (0⋅77–0⋅83)	0⋅79 (0⋅73–0⋅85)
Adjusted	0⋅86 (0⋅81–0⋅93)	0⋅95 (0⋅90–0⋅99)	0⋅84 (0⋅74–0⋅95)	0⋅90 (0⋅86–0⋅95)	0⋅90 (0⋅83–0⋅97)

For diabetes: adjusted for thiamine intake, age group, sex, elevated waist circumstances, education level, family history of hyperlipidemia, diabetes, smoking status, monthly household income, BMI group, dyslipidemia and occupation.

For hypertension: adjusted for thiamine intake, elevated waist circumstances, sex, and age group, education level, residential areas, monthly household income, diabetes, BMI groups, family history of diabetes or CVDs and dyslipidemia.

For myocardial infarction or angina: adjusted for thiamine intake, sex, and age group, education level, dyslipidemia, diabetes, elevated waist circumstances and family history of CVDs.

For dyslipidemia: adjusted for thiamine intake, occupation, sex, education level, high-risk drinking, family history of hyperlipidemia, elevated waist circumference and BMI group.

For depression: adjusted for thiamine intake, age group, sex, occupation, education level, physical activity, smoking status and BMI group.

**Fig. 3.**
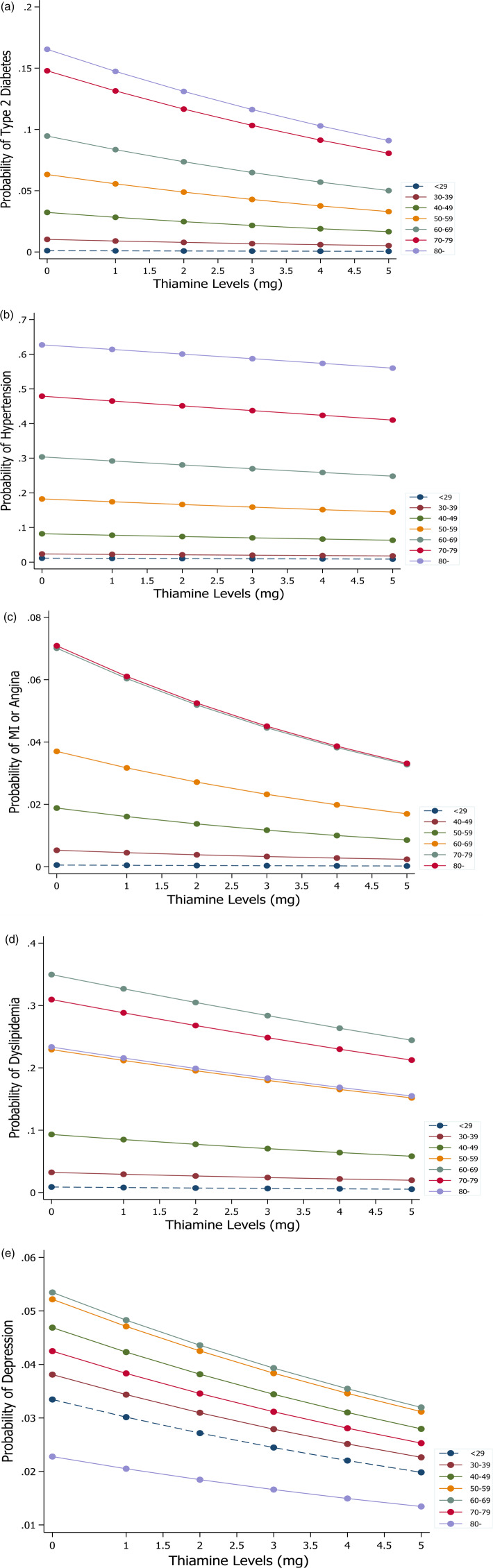
Marginal effects of thiamine intake on (a) type 2 diabetes, (b) hypertension, (c) myocardial or angina, (d) dyslipidemia and (e) depression by age group.

Figure 3 shows the marginal effects of thiamine intake on hypertension, MI or angina, type 2 diabetes, depression and dyslipidemia by age. Risks were found to diminish as thiamine intake increased. Of note, the probabilities of stroke and diabetes decreased rapidly among older participants as thiamine intake levels increased by 1 mg.

## Discussion

This large-scale study is the first to report the association between dietary thiamine intake and the prevalence of CVDs, diabetes and mental health issues at the national level in Korea. The study also provides thiamine dietary consumption trends over recent years and contributes to understanding of the effect that thiamine consumption has on the Korean population.

The present study shows that thiamine intake is significantly and negatively associated with the risk of type 2 diabetes. In addition, HbA1c and fasting glucose levels were lower in the sufficient thiamine intake group than in the insufficient intake group. Our findings support those of a previous study, in which a substantial decrease in plasma fasting glucose concentration was observed in drug-naïve patients with type 2 diabetes administered 150 mg of thiamine daily for 1 month^(16)^. Although the relationship between type 2 diabetes and thiamine deficiency has not been fully elucidated, reduced thiamine supply in diabetic vascular cells has been reported to exacerbate metabolic dysfunction under hyperglycemic conditions^(17)^, and thiamine deficiency was found to contribute to severe insulin synthesis and secretion dysfunction in human cell lines^(18)^. Distal nephrons excrete excess thiamine that is not bound to protein, and thiamine loss is closely related to renal clearance^(19)^, thus, increased renal clearance due to type 2 diabetes may be linked to thiamine deficiency. In addition, intestinal motility affected by autonomic neuropathy in type 2 diabetes causes overgrowth of bacteria in the small intestine and inhibits thiamine absorption^(20)^.

Our results also show a significant decrease in total serum cholesterol among individuals with sufficient thiamine intake. The percentages of participants with elevated total cholesterol and LDL-C levels were lower in the sufficient thiamine intake group and the percentage with a low HDL-C level was lower in this group, which is consistent with previous findings^(21)^. Furthermore, we found that thiamine intake had a significant impact on the development of CVDs such as hypertension, MI or angina, which supports the notion that thiamine ameliorates the detrimental effect of elevated endothelial glucose by reducing the glycation of intracellular proteins^(22)^. Thiamine also plays a fundamental role in the prevention of atherosclerotic plaque because it has a defensive effect on the impact of glucose- and insulin-mediated proliferation on human infragenicular arterial smooth muscle cells^(23)^. Our data support reports that regular thiamine administration boosts endothelial functions and retards atherosclerosis progression^(24)^, which is in-line with a report that short-term thiamine therapy regenerated endothelial function in healthy smokers with endothelial dysfunction triggered by smoking^(25)^.

Notably, we found that thiamine intakes can reversal the risks of depression. This concurs with the findings of cross-sectional studies performed in China and the United Kingdom, which showed low thiamine serum concentrations were associated with high prevalence of depression symptoms^(26,27)^. Another study also showed that the short-term daily thiamine administration prompted a feeling of well-being in the elderly and enhanced the energetic statuses and ‘clear-headedness’ of young women^(28,29)^, which may be because oxidative stress caused by thiamine deficiency reduces hippocampal volume and causes neural damage in depressed patients^(30,31)^. Besides, thiamine is an important coenzyme during the syntheses of many neurotransmitters, such as acetylcholine, aspartate, serotonin and glutamate, and deficiencies in the functions of these neurotransmitters, and thus, thiamine deficiency would be expected to result in symptoms like depression or stress^(32)^.

Importantly, the present study raises a public health concern for people in Korea. The average thiamine intakes reported in Korea decreased from 2014 to 2018. It could be explained that rapid changes in diets and lifestyles that have taken place because of urbanisation, industrialisation, economic development and globalisation, and these have changed dietary patterns towards high-saturated, high-fat and low-energy-dense diet^(1,2)^. Furthermore, rapid industrialisation has led to an increase in the number of nuclear families and single households^(33)^. Of note, eating alone is related to an unhealthy dietary intake, including more fried foods or carbonated beverages, and less fruits and vegetables^(34)^. On the other hand, the majority of participants in the present study located in urban areas (especially in 2014 and 2015); therefore, they were more likely to be used fat foods and consumed less fruits and vegetables. Our findings were consistent with the previous studies^(34–36)^.

Thirteen dietary surveys in nine countries of the European Union reported average thiamine intakes between 0⋅31 and 0⋅65 mg/d, and the present study shows that thiamine intake in Korea was lower than that in the United States in adult aged ≥20 years (1⋅76 (1⋅07) mg (male), 1⋅34 (0⋅79) mg *v.* 1⋅95 mg (male), 1⋅39 (women)). Furthermore, the present study also shows the average whole population thiamine intake was lower than that in Spain (1⋅17 ± 0⋅02 mg/d, from 0⋅30 to 3⋅44 mg/d)^(37)^. Of note, thiamine intakes in Korean women in 2016–18 were 1⋅126, 1⋅115 and 1⋅087 mg, which were equal to or only slightly above the recommended intake of 1⋅10 mg/d for women^(38)^. On the other hand, the average daily intake of thiamine among children aged 1 to <3 years old (0⋅58–0⋅98 mg/d), aged 3 to <10 years old (0⋅68–1⋅29 mg/d), aged 10 to <18 years old (0⋅93–1⋅92 mg/d) and among aged ≥18 years old (0⋅88–1⋅99 mg/d) in nine countries of the European Union was higher than the present study^(39)^. The average daily intake of thiamine in the United States followed a similar pattern. The average daily intake of thiamine is 1⋅51 mg for 2–5 years, 1⋅76 mg for 6–11 years, 1⋅95 mg in 12–19 years of age and adults aged 20 and older is 4⋅89 mg in men and 4⋅90 mg in women^(40)^. In the present study, the average daily intake of thiamine among adults aged ≥65 living in the rural and urban areas was 1⋅53 mg, 1⋅58 mg in men and 1⋅17 mg, 1⋅19 mg in women, which was higher than a study in fifteen provinces (autonomous regions and municipalities) in China (0.80 mg in men and 0.70 mg in women^(41)^). Given the steady decrease in thiamine intake observed in the present study and the observed beneficial effects of thiamine in the elderly on the risks of stroke, diabetes, depression and stress, we suggest health policymakers and public health practitioners invest in prevention and control plans.

The present study has some limitations that deserve consideration. First, the cross-sectional design of the study prevented evaluations of causality between NCDs and mental health and thiamine intakes. Second, since no physiological markers of antioxidant status were measured in KNHANES, oxidation status and thiamine levels in plasma and tissues were not evaluated. Third, thiamine intake was calculated based on 24-h recall data and intake amounts may have differed on weekdays and weekends, and thus, intakes may have been under- or overestimated. However, 24-h recall offers a cost-effective means of assessing food intakes, and our findings show a significant decreasing trend in thiamine use over recent years.

Our findings showed that the trend in the levels of thiamine intake tends to be decreasing in recent years. It is crucial to develop a prevention strategy targeting the population to slow down this progression to postpone risk factors related to insufficient thiamine intake and reduce prevalence. The present study confirmed the role of thiamine in the reversal of NCDs and mental health and risk factors associated with its deficiency. These findings highlight the value of urgent efforts to establish targeted thiamine supplementation in Korea. We believe that these strategies would effectively reduce the prevalence of NCDs and mental health.

## Supplementary material

For supplementary material accompanying this paper visit http://dx.doi.org/10.1017/jns.2021.23. click here to view supplementary material
