# Supplementary material for: Association between levels of thiamine intake, diabetes, cardiovascular diseases and depression in Korea: a national cross-sectional study
Source: J Nutr Sci. 2021 Apr 27;10:e31. doi: 10.1017/jns.2021.23 (PMC8141681; doi:10.1017/jns.2021.23)
Supplement: Supplementary file 1 [file S2048679021000239sup001.docx]

**Supplementary material**

**Table S1.** The relationship between thiamine intake and CVDs, type 2 diabetes, dyslipidemia, and mental health.

| **Variables** | **Thiamine intake** | | **Crude Odds ratio** † **95%Cl** | ***p*-value** |
| --- | --- | --- | --- | --- |
|  | **Insufficiency** | **Sufficiency** |  |  |
| **Hypertension** (n=31923) |  |  |  |  |
| *Yes* | 2630 (21.5) | 3704 (18.8) | 0.84 (0.80 — 0.89) | <0.001 |
| *No* | 9599 (78.5) | 15990 (81.2) |  |  |
| **MI and angina** (n= 31185) |  |  |  |  |
| *Yes* | 334 (2.8) | 410 (2.1) | 0.75 (0.65 — 0.87) | <0.001 |
| *No* | 11553 (97.2) | 18888 (97.9) |  |  |
| **MI** (n= 31188) |  |  |  |  |
| *Yes* | 122 (1.0) | 155 (0.8) | 0.78 (0.62 — 0.99) | 0.042 |
| *No* | 11767 (99.0) | 19144 (99.2) |  |  |
| **Angina** (n= 31185) |  |  |  |  |
| *Yes* | 234 (2.0) | 292 (1.5) | 0.77 (0.64 — 0.91) | 0.002 |
| *No* | 11653 (98.0) | 19006 (98.5) |  |  |
| **Stroke** (n= 31194) |  |  |  |  |
| *Yes* | 298 (2.5) | 331 (1.7) | 0.68 (0.58 — 0.80) | <0.001 |
| *No* | 11593 (97.5) | 18972 (98.3) |  |  |
| **Dyslipidemia** (n= 26814) |  |  |  |  |
| *Yes* | 1786 (18.9) | 2646 (15.2) | 0.77 (0.72 — 0.83) | <0.001 |
| *No* | 7671 (81.1) | 14711 (84.8) |  |  |
| **Type 2 diabetes** (n= 31910) |  |  |  |  |
| *Yes* | 1079 (8.8) | 1362 (6.9) | 0.77 (0.71 — 0.83) | <0.001 |
| *No* | 11142 (91.2) | 18327 (93.1) |  |  |
| **Depression** (n= 31175) |  |  |  |  |
| *Yes* | 525 (4.4) | 674 (3.5) | 0.78 (0.70 — 0.88) | <0.001 |
| *No* | 11357 (95.6) | 18619 (96.5) |  |  |
| **Stress** (n= 27500) |  |  |  |  |
| *Yes* | 2628 (27.3) | 4525 (25.3) | 0.90 (0.85 — 0.95) | <0.001 |
| *No* | 6985 (72.7) | 13362 (74.7) |  |  |

† Reference: the insufficient thiamine intake group. *p*-value was analyzed by binary logistic regression.

**Table S2.** Adjusted ORs for hypertension.

| **Variables** | **Odds Ratio** | **Std.Err.** | **z** | ***P*-value** | **95% CI** | |
| --- | --- | --- | --- | --- | --- | --- |
| **Thiamine intake** *(mg)* | 0.945 | 0.024 | -2.220 | 0.026 | 0.898 | 0.993 |
| **Elevated waist circumstances** *(%)* |  |  |  |  |  |  |
| *No* | 1 (refer) |  |  |  |  |  |
| *yes* | 1.587 | 0.087 | 8.410 | <0.001 | 1.425 | 1.768 |
| **Age group** *(%)* |  |  |  |  |  |  |
| *<29* | 1 (refer) |  |  |  |  |  |
| *30-39* | 1.959 | 0.477 | 2.760 | 0.006 | 1.216 | 3.156 |
| *40-49* | 7.132 | 1.569 | 8.930 | <0.001 | 4.634 | 10.977 |
| *50-59* | 17.759 | 3.848 | 13.280 | <0.001 | 11.614 | 27.155 |
| *60-69* | 34.240 | 7.447 | 16.250 | <0.001 | 22.356 | 52.439 |
| *70-79* | 71.748 | 15.822 | 19.380 | <0.001 | 46.568 | 110.541 |
| *≥80* | 130.932 | 31.262 | 20.420 | <0.001 | 81.999 | 209.066 |
| **Sex** *(%)* |  |  |  |  |  |  |
| *Male* | 1 (refer) |  |  |  |  |  |
| *Female* | 0.551 | 0.026 | -12.480 | <0.001 | 0.502 | 0.606 |
| **Education level** *(%)* |  |  |  |  |  |  |
| *≤ Middle school* | 1 (refer) |  |  |  |  |  |
| *High school* | 0.808 | 0.045 | -3.850 | <0.001 | 0.724 | 0.900 |
| *≥ College* | 0.669 | 0.044 | -6.190 | <0.001 | 0.589 | 0.760 |
| **Monthly household income** *(%)* |  |  |  |  |  |  |
| *< 2,000* | 1 (refer) |  |  |  |  |  |
| *≥ 2,000 and < 4,000* | 0.864 | 0.049 | -2.600 | 0.009 | 0.774 | 0.965 |
| *≥ 4,000 and < 6,000* | 0.765 | 0.052 | -3.970 | <0.001 | 0.670 | 0.873 |
| *≥ 6,000* | 0.807 | 0.055 | -3.130 | 0.002 | 0.706 | 0.923 |
| **Type 2 diabetes** *(%)* |  |  |  |  |  |  |
| *No* | 1 (refer) |  |  |  |  |  |
| *yes* | 2.425 | 0.155 | 13.840 | <0.001 | 2.139 | 2.749 |
| **Family history of diabetes** (%) |  |  |  |  |  |  |
| *No* | 1 (refer) |  |  |  |  |  |
| *yes* | 0.802 | 0.042 | -4.180 | <0.001 | 0.724 | 0.890 |
| **BMI group** *(%)* |  |  |  |  |  |  |
| *<18.5* | 1 (refer) |  |  |  |  |  |
| 25-30 | 1.589 | 0.087 | 8.430 | <0.001 | 1.426 | 1.769 |
| >30 | 3.038 | 0.309 | 10.930 | <0.001 | 2.489 | 3.708 |
| **Dyslipidemia** *(%)* |  |  |  |  |  |  |
| *No* | 1 (refer) |  |  |  |  |  |
| *yes* | 2.940 | 0.141 | 22.450 | <0.001 | 2.676 | 3.231 |
| **Family history of CVDs** (%) |  |  |  |  |  |  |
| *No* | 1 (refer) |  |  |  |  |  |
| *yes* | 2.593 | 0.118 | 20.960 | <0.001 | 2.372 | 2.834 |
| LR chi2(20) = 7380.12 Prob > chi2 = 0.0000 Log likelihood = -7105.8204 Pseudo R2 = 0.3418 | | | | | | |

**Table S3.** Adjusted ORs for myocardial infraction or angina.

| **Variables** | **Odds Ratio** | **Std.Err.** | **z** | ***P*-value** | **95% CI** | |
| --- | --- | --- | --- | --- | --- | --- |
| **Thiamine intake** *(mg)* | 0.838 | 0.055 | -2.720 | 0.007 | 0.737 | 0.952 |
| **Dyslipidemia** (%) |  |  |  |  |  |  |
| *No* | 1 (refer) |  |  |  |  |  |
| *Yes* | 2.400 | 0.235 | 8.960 | <0.001 | 1.982 | 2.907 |
| **Age group** (%) |  |  |  |  |  |  |
| *<29* | 1 (refer) |  |  |  |  |  |
| *40-49* | 8.254 | 8.517 | 2.050 | 0.041 | 1.092 | 62.376 |
| *50-59* | 30.159 | 30.407 | 3.380 | 0.001 | 4.180 | 217.583 |
| *60-69* | 66.345 | 66.694 | 4.170 | <0.001 | 9.250 | 475.862 |
| *70-79* | 143.588 | 144.278 | 4.940 | <0.001 | 20.037 | 1028.984 |
| *≥80* | 153.240 | 155.560 | 4.960 | <0.001 | 20.955 | 1120.630 |
| **Sex** *(%)* |  |  |  |  |  |  |
| *Male* | 1 (refer) |  |  |  |  |  |
| *Female* | 0.408 | 0.042 | -8.710 | <0.001 | 0.333 | 0.499 |
| **Family history of CVDs** (%) |  |  |  |  |  |  |
| *No* | 1 (refer) |  |  |  |  |  |
| *yes* | 1.702 | 0.163 | 5.570 | <0.001 | 1.411 | 2.052 |
| **Education level** *(%)* |  |  |  |  |  |  |
| *≤ Middle school* | 1 (refer) |  |  |  |  |  |
| >= college | 0.755 | 0.103 | -2.060 | 0.039 | 0.578 | 0.986 |
| **Type 2 diabetes** *(%)* |  |  |  |  |  |  |
| *No* |  |  |  |  |  |  |
| *yes* | 1.289 | 0.143 | 2.280 | 0.022 | 1.036 | 1.602 |
| **Elevated waist circumstances** *(%)* |  |  |  |  |  |  |
| *No* | 1 (refer) |  |  |  |  |  |
| *yes* | 1.322 | 0.130 | 2.850 | 0.004 | 1.091 | 1.602 |
| LR chi2(12) = 788.25 Prob > chi2 = 0.0000 Log likelihood = -1930.9385 Pseudo R2 = 0.1695 | | | | | | |

**Table S4.** Adjusted ORs for dyslipidemia.

| **Variables** | **Odds Ratio** | **Std.Err.** | **z** | ***P*-value** | **95% CI** | |  |
| --- | --- | --- | --- | --- | --- | --- | --- |
| **Thiamine intake** *(mg)* | 0.901 | 0.023 | -4.060 | <0.001 | 0.857 | 0.948 |  |
| **Occupation** *(%)* |  |  |  |  |  |  |  |
| *Managers, professional* | 1 (refer) |  |  |  |  |  |  |
| *Office worker, clerical workers* | 0.818 | 0.073 | -2.250 | 0.025 | 0.687 | 0.975 |  |
| *Service workers, sales workers* | 0.874 | 0.057 | -2.060 | 0.040 | 0.768 | 0.994 |  |
| *Agriculture, forestry and fishing workers* | 0.662 | 0.057 | -4.770 | <0.001 | 0.559 | 0.784 |  |
| **Age group** (%) |  |  |  |  |  |  |  |
| <29 | 1 (refer) |  |  |  |  |  |  |
| 30-39 | 3.305 | 0.798 | 4.950 | <0.001 | 2.058 | 5.306 |  |
| 40-49 | 10.035 | 2.282 | 10.140 | <0.001 | 6.426 | 15.669 |  |
| 50-59 | 28.964 | 6.494 | 15.010 | <0.001 | 18.664 | 44.948 |  |
| 60-69 | 52.855 | 11.912 | 17.600 | <0.001 | 33.982 | 82.210 |  |
| 70-79 | 45.112 | 10.311 | 16.670 | <0.001 | 28.824 | 70.606 |  |
| ≥80 | 30.807 | 7.640 | 13.820 | <0.001 | 18.948 | 50.088 |  |
| **Sex** *(%)* |  |  |  |  |  |  |  |
| *Male* | 1 (refer) |  |  |  |  |  |  |
| *Female* | 1.218 | 0.057 | 4.250 | <0.001 | 1.112 | 1.334 |  |
| **Education level** *(%)* |  |  |  |  |  |  |  |
| *≤ Middle school* | 1 (refer) |  |  |  |  |  |  |
| *High school* | 0.894 | 0.048 | -2.110 | 0.035 | 0.805 | 0.992 |  |
| *≥ College* | 0.851 | 0.052 | -2.650 | 0.008 | 0.755 | 0.959 |  |
| **High risk drinking** (%) |  |  |  |  |  |  |  |
| *No* | 1 (refer) |  |  |  |  |  |  |
| *Yes* | 0.716 | 0.064 | -3.770 | <0.001 | 0.602 | 0.852 |  |
| **Family history of hyperlipidemia** (%) |  |  |  |  |  |  |  |
| *No* | 1 (refer) |  |  |  |  |  |  |
| *Yes* | 2.961 | 0.224 | 14.320 | <0.001 | 2.553 | 3.436 |  |
| **BMI group** *(%)* |  |  |  |  |  |  |  |
| *<18.5* | 1 (refer) |  |  |  |  |  |  |
| *≥ 18.5 and < 25* | 2.449 | 0.470 | 4.670 | <0.001 | 1.682 | 3.567 |  |
| *≥ 25 and < 30* | 2.960 | 0.585 | 5.490 | <0.001 | 2.009 | 4.360 |  |
| *≥ 30* | 4.198 | 0.895 | 6.730 | <0.001 | 2.764 | 6.376 |  |
| **Elevated waist circumstances** *(%)* |  |  |  |  |  |  |  |
| *No* | 1 (refer) |  |  |  |  |  |  |
| *Yes* | 1.631 | 0.086 | 9.270 | <0.001 | 1.471 | 1.809 |  |
| LR chi2(19) = 3435.31 Prob > chi2 = 0.0000 Log likelihood = -7698.8543 Pseudo R2 = 0.1824 | | | | | | | |

**Table S5.** Adjusted ORs for depression.

| **Variables** | **Odds Ratio** | **Std.Err.** | **z** | ***P*-value** | **95% CI** | |
| --- | --- | --- | --- | --- | --- | --- |
| **Thiamine intake** *(mg)* | 0.898 | 0.035 | -2.740 | 0.006 | 0.831 | 0.970 |
| **Monthly household income** *(%)* |  |  |  |  |  |  |
| *< 2,000* | 1 (refer) |  |  |  |  |  |
| *≥ 2,000 and < 4,000* | 0.651 | 0.053 | -5.290 | <0.001 | 0.555 | 0.763 |
| *≥ 4,000 and < 6,000* | 0.500 | 0.051 | -6.750 | <0.001 | 0.409 | 0.612 |
| *≥ 6,000* | 0.589 | 0.059 | -5.300 | <0.001 | 0.484 | 0.716 |
| **Age group** (%) |  |  |  |  |  |  |
| *<29* | 1 (refer) |  |  |  |  |  |
| *40-49* | 1.312 | 0.144 | 2.470 | 0.013 | 1.058 | 1.627 |
| *50-59* | 1.514 | 0.158 | 3.970 | <0.001 | 1.233 | 1.858 |
| *60-69* | 1.601 | 0.168 | 4.490 | <0.001 | 1.304 | 1.965 |
| *70-79* | 1.280 | 0.148 | 2.130 | 0.033 | 1.020 | 1.607 |
| *≥80* | 0.678 | 0.123 | -2.140 | 0.032 | 0.475 | 0.967 |
| **Sex** *(%)* |  |  |  |  |  |  |
| *Male* | 1 (refer) |  |  |  |  |  |
| *Female* | 2.714 | 0.229 | 11.830 | <0.001 | 2.301 | 3.203 |
| **Occupation** *(%)* |  |  |  |  |  |  |
| *Managers, professional* | 1 (refer) |  |  |  |  |  |
| *Elementary occupations* | 1.357 | 0.155 | 2.670 | 0.008 | 1.084 | 1.698 |
| *Unemployed* | 1.994 | 0.148 | 9.310 | <0.001 | 1.724 | 2.306 |
| **Education level** *(%)* |  |  |  |  |  |  |
| *≤ Middle school* | 1 (refer) |  |  |  |  |  |
| *>= college* | 0.732 | 0.063 | -3.620 | <0.001 | 0.619 | 0.867 |
| **Physical activity** *(%)* |  |  |  |  |  |  |
| *Not regular* | 1 (refer) |  |  |  |  |  |
| *Regular* | 0.842 | 0.066 | -2.190 | 0.028 | 0.722 | 0.982 |
| **Smoking status** *(%)* |  |  |  |  |  |  |
| *Current smoker* | 1 (refer) |  |  |  |  |  |
| *Non/ex-smoker* | 1.706 | 0.165 | 5.530 | <0.001 | 1.412 | 2.061 |
| **BMI group** *(%)* |  |  |  |  |  |  |
| *<18.5* | 1 (refer) |  |  |  |  |  |
| 18.5-25 | 0.764 | 0.076 | -2.710 | 0.007 | 0.629 | 0.929 |
| 25-30 | 0.798 | 0.086 | -2.100 | 0.036 | 0.646 | 0.985 |
| LR chi2(17) = 641.43 Prob > chi2 = 0.0000  Log likelihood = -4361.5601 Pseudo R2 = 0.0685 | | | | | | |

**Table S6.** Adjusted ORs for type 2 diabetes.

| **Variables** | **Odds Ratio** | **Std.Err.** | | **z** | ***P*-value** | **95% CI** | |
| --- | --- | --- | --- | --- | --- | --- | --- |
| **Thiamine intake** *(mg)* | 0.868 | 0.032 | | -3.820 | <0.001 | 0.808 | 0.934 |
| **Age group** (%) |  |  | |  |  |  |  |
| <29 | 1 (refer) |  | |  |  |  |  |
| 30-39 | 10.181 | 7.455 | | 3.170 | 0.002 | 2.423 | 42.769 |
| 40-49 | 33.445 | 23.890 | | 4.910 | <0.001 | 8.247 | 135.632 |
| 50-59 | 69.511 | 49.434 | | 5.960 | <0.001 | 17.247 | 280.156 |
| 60-69 | 111.104 | 78.953 | | 6.630 | <0.001 | 27.596 | 447.317 |
| 70-79 | 192.110 | 136.638 | | 7.390 | <0.001 | 47.658 | 774.398 |
| ≥80 | 220.863 | 158.669 | | 7.510 | <0.001 | 54.027 | 902.884 |
| **Sex** *(%)* |  |  | |  |  |  |  |
| *Male* | 1 (refer) |  | |  |  |  |  |
| *Female* | 0.441 | 0.030 | | -12.200 | <0.001 | 0.386 | 0.503 |
| **Elevated waist circumstances** *(%)* |  |  | |  |  |  |  |
| *No* | 1 (refer) |  | |  |  |  |  |
| *yes* | 1.715 | 0.104 | | 8.910 | <0.001 | 1.523 | 1.932 |
| **Education level** *(%)* |  |  | |  |  |  |  |
| *≤ Middle school* | 1 (refer) |  | |  |  |  |  |
| *>= college* | 0.646 | 0.052 | | -5.380 | <0.001 | 0.551 | 0.758 |
| **Family history of hyperlipidemia** (%) |  |  | |  |  |  |  |
| *No* | 1 (refer) |  | |  |  |  |  |
| *Yes* | 0.683 | 0.093 | | -2.800 | 0.005 | 0.523 | 0.892 |
| **Family history of diabetes** (%) |  |  | |  |  |  |  |
| *No* | 1 (refer) |  | |  |  |  |  |
| *Yes* | 3.435 | 0.210 | | 20.160 | <0.001 | 3.047 | 3.873 |
| **Smoking status** *(%)* |  |  | |  |  |  |  |
| *Current smoker* | 1 (refer) |  | |  |  |  |  |
| *Non/ex-smoker* | 1.237 | 0.102 | | 2.580 | 0.010 | 1.053 | 1.454 |
| **Monthly household income** *(%)* |  |  | |  |  |  |  |
| *< 2,000* | 1 (refer) |  | |  |  |  |  |
| *≥ 4,000 and < 6,000* | 0.818 | 0.070 | | -2.350 | 0.019 | 0.692 | 0.967 |
| *≥ 6,000* | 0.808 | 0.071 | | -2.450 | 0.014 | 0.680 | 0.958 |
| **BMI group** *(%)* |  |  | |  |  |  |  |
| *<18.5* | 1 (refer) |  | |  |  |  |  |
| >30 | 1.450 | 0.168 | | 3.220 | 0.001 | 1.156 | 1.819 |
| **Dyslipidemia** (%) |  |  | |  |  |  |  |
| *No* | 1 (refer) |  | |  |  |  |  |
| *Yes* | 3.274 | 0.193 | | 20.120 | <0.001 | 2.917 | 3.675 |
|  | | |  |  |  |  |  |
| **Occupation** (%) |  |  | |  |  |  |  |
| *Managers, professional* | 1 (refer) |  | |  |  |  |  |
| *Unemployed* | 1.261 | 0.079 | | 3.690 | <0.001 | 1.115 | 1.427 |
| LR chi2(18) = 2913.99 Prob > chi2 = 0.0000  Log likelihood = -4438.6884 Pseudo R2 = 0.2471 | | | | | | | |
